# Quality of life of survivors following road traffic orthopaedic injuries in Rwanda

**DOI:** 10.3389/fpubh.2024.1405697

**Published:** 2024-07-17

**Authors:** J. C. Allen Ingabire, David K. Tumusiime, Jean Baptiste Sagahutu, Gerard Urimubenshi, Georges Bucyibaruta, Sonti Pilusa, Aimee Stewart

**Affiliations:** ^1^Department of Surgery, University Teaching Hospital of Kigali, University of Rwanda, Kigali, Rwanda; ^2^Physiotherapy Department, University of Rwanda, Kigali, Rwanda; ^3^Department of Epidemiology and Biostatistics, Imperial College London, London, United Kingdom; ^4^Physiotherapy Department, University of the Witwatersrand, Johannesburg, South Africa

**Keywords:** disability, EQ-5D-5L, quality of life, road traffic orthopaedic injuries, Rwanda

## Abstract

**Background:**

Road traffic injuries (RTI) pose a global public health threat, especially in low- and middle-income nations. These injuries typically cause orthopaedic problems that may negatively impair a person’s physical and mental health and quality of life. Our study examined the quality of life of road traffic orthopaedic injuries (RTOI) survivors.

**Methods:**

A cross-sectional study at five Rwandan referral hospitals, included 369 adult RTOI victims. Two years post-injury, participants completed the European Quality of life 5 Dimension 5 (EQ-5D-5L) and Visual Analogue Scale (VAS) Questionnaire between June 2 and August 31, 2022, with informed consent. Three EQ-5D-5L-VAS scores were used: low (0–40%), fair (41–60%), and excellent (61–100%). We used logistic regression analysis with a significance threshold of *p* < 0.05 to determine odds ratios (OR) and 95% CI.

**Results:**

The RTOI victims had a mean age of 37.5 ± 11.26 years with sex ratio M:F:3:1. Usual activities (66.8%) and mobility (54.8%) were the most affected EQ-5D-5L dimensions. Residence, hospital stay, rehabilitation, and return to work affected mobility, usual activities, pain/discomfort, and anxiety/depression. The EQ-5D-5L/VAS score showed 34.95% poor QoL (0–40%) and 35.50% good QoL. Factors affecting QoL include level of education (OR = 1.66, *p* < <0.01), type of intervention (OR = 1.22, *p* = 0.003), rehabilitation (OR = 2.41, *p* < 0.01) and level of disability (OR = 196.41, *p* < 0.01). Mobility, self-care, usual activities, pain, comfort, anxiety, and depression vary moderately on Shannon’s index.

**Conclusion:**

The study highlights the significant impact of road traffic orthopaedic injuries (RTOI) on survivors’ quality of life in Rwanda, revealing challenges in mobility and daily activities. Factors influencing quality of life include education level, medical intervention type, rehabilitation, and disability degree. The findings emphasize the need for tailored rehabilitation strategies and policy interventions to improve long-term outcomes for RTOI survivors.

## Background

Road traffic injuries (RTIs) are a significant global public health issue, causing substantial physical, psychological, and social consequences for individuals involved (1). Each year, approximately 50 million people are involved in road traffic injuries globally, resulting in 1.2 million fatalities. Additionally, 30% of survivors endure permanent disabilities, while 14% are unable to resume work (2). Survivors of RTIs often experience long-term impairments and disabilities that can affect their overall quality of life (QoL) (3). Assessing the QoL outcomes of RTI survivors is crucial for understanding the impact of these injuries and developing appropriate interventions to enhance survivors’ well-being and social reintegration (4).

Research has demonstrated that achieving positive results for these injuries relies on prompt medical attention, including accurate diagnosis and suitable surgical treatment, as well as thorough postoperative monitoring that includes rehabilitation, as well as social and economic assistance. However, these aspects remain difficult to implement in many low- and middle-income countries (LMICs) (5). Lack of sufficient and effective medical attention following an injury has a detrimental effect on the victims’ ability to function and their overall quality of life. This is a significant problem in low- and middle-income countries (LMICs) (6–8).

Studies to investigate the QoL of RTI typically utilize validated QoL assessment tools to measure different domains such as physical functioning, psychological well-being, social interactions, and overall life satisfaction (9). The findings from these studies provide insights into the challenges faced by RTI survivors, the effectiveness of healthcare interventions, and the need for support services to improve their Health-Related Quality of Life (HRQoL) (10).

As per international figures, disabilities arising from road traffic injuries are costly for both society and individuals. The annual costs of road traffic injuries in low and middle-income countries are estimated to be between US$65–100 billion, more than the total yearly amount received in development aid affecting both the wellbeing of the victims and their families (11, 12). In 2011, a study conducted on motorcycles injuries in Rwanda estimated the individual and social cost to be around1.3$ million without counting the long-term disabilities’ costs (13).

In 2019, the Rwanda National Police documented a total of 4,661 injuries and 700 deaths resulting from RTI. For these injuries, half had orthopaedic problems, 35.6% permanent disabilities and 36% of victims were unable to return to work. Age, gender, socioeconomic status, the severity of the injury, rehabilitation, and hospital length of stay affect the level of disability and social reintegration of the road traffic orthopaedic injuries in Rwanda (14, 15).

Currently, the predominant emphasis in systematic data collecting is in the acute phase of trauma care, namely within the confines of a patient’s duration of stay in the trauma centre. The gathering of data regarding post-discharge outcomes is predominantly limited to small studies (16). Managing these injuries poses significant challenges for both the physician and the nursing team, who must provide continuous care for the patients (17, 18). This constraint impedes our comprehension of the potential impact of acute care adjustments on long-term functional outcomes and quality of life for individuals who have experienced trauma (16). The existence of this data gap is particularly widespread in numerous countries, particularly in Low and Middle-Income nations (LMICs).

Orthopaedic injuries resulting from road traffic accidents can lead to long-term physical, psychological, and socioeconomic consequences for survivors (19). The quality of life of survivors following road traffic orthopaedic injuries in Rwanda is an important aspect to consider in understanding the impact of such injuries on individuals and society. Our study aimed to determine the quality of life of survivors following Road Traffic Orthopaedic Injuries (RTOI) in Rwanda.

## Methodology

### Study design and sample size

A cross-sectional study on orthopaedic injuries conducted from June 2 to August 31, 2022, included participants 2 years post-injury resulting from road traffic accidents in Rwanda. The study analysed hospital data from five referral hospitals in 2019. Referral and teaching hospitals provide emergency care, orthopaedic services, mental health treatment, and rehabilitation. The study sites included were Centre Hospitalier Universitaire de Kigali (CHUK), Rwanda Military Hospital (RMH), King Faisal Hospital (KFH) in Kigali City, Centre Hospitalier Universitaire (CHUB) in the Southern Province, and Ruhengeri Hospital (RH) in the Northern Province.

We recorded 4,600 post-RTI cases, 1,986 of whom were adult orthopaedic injuries, and we sampled 369 representative RTI victims using Krejcie and Morgan’s formula (20). To collect patient data, all hospital records were carefully examined. The study included adult patients (18 years and older) who had orthopaedic injuries from road traffic accidents and were 2 years post-injury, treated at five Rwandan referral hospitals. Participants had to give informed consent and be classified using the Kampala Trauma Score. Exclusions were patients without orthopaedic injuries, those younger than 18, less than 2 years post-injury, lacking informed consent, or with non-compliant hospital records. The remaining qualified individuals were phoned at their homes for demographic information and invited to the hospital for further assessment.

### Psychometric properties of the instruments

The EQ-5D-5L is an instrument that evaluates the generic quality of life and was developed in Europe by the EuroQol group. It has been validated in many countries and languages. The EQ-5D-5L demonstrates robust psychometric properties, with studies showing a mean HRQoL score of 0.79 (SD 0.17) and a mean EQ-VAS score of 71.7 (SD 19.4). It effectively detects meaningful health changes, avoiding ceiling effects seen in the EQ-5D-3L. Reliability is confirmed with significant test–retest correlations, and construct validity is supported by correlations with related health measures. The instrument is sensitive across sociodemographic groups, with younger and higher-educated individuals reporting better HRQoL scores (21–23). Permission to use the EQ-5D-5L questionnaire in our study was obtained from the EuroQol group with registration ID: 48658.It is used widely as a self-assessed health-related quality of life questionnaire with five components: mobility, self-care, usual activities, pain/discomfort, and anxiety/depression. It also has five levels, where each one is rated on a scale that describes the degree of problems in that area. The EQ-5D-5L measures five levels: Level 1 indicates no issues, Level 2 minor, Level 3 moderate, Level 4 severe, and Level 5 extreme problems or inability to operate.

The EQ-5D-5L is used in conjunction with EQ-VAS, a visual analogue scale, evaluating self-reported patient general well-being, rated at percentages, from worst, equal to 0 to best, which is equivalent to 100.It is a validated tool for economic evaluation, clinical studies, quality of care, and public health studies (24). The EQ-5D-5L questionnaire underwent rigorous translation by two language experts who translated it from English to Kinyarwanda and back to English to ensure cultural and linguistic equivalence. Orthopedic and rehabilitation experts then reviewed the translation for quality, clarity, and appropriateness for Rwandan participants. This comprehensive feedback process enhanced the questionnaire’s reliability and comprehensibility, leading to higher participant engagement and accurate data collection on quality-of-life dimensions. Consequently, the translated questionnaire provided reliable data for healthcare interventions and allowed for comparability with international studies.

The EQ-5D-5L VAS scores range from 0 to 100 but are often grouped for easier analysis and interpretation. The scores are categorized as Very Poor (0–20), Poor (3, 10, 21–38), Fair (41–60), Good (61–80), and Very Good (81–100). In our study, the ‘Very Poor’ category was merged with the ‘Poor’ category to form a consolidated ‘Poor’ category. The category labelled as ‘Fair’ remained unchanged. Furthermore, we consolidated the ‘Good’ and ‘Very Good’ categories into a singular ‘Good’ category.

The WHODAS 2.0 is a multi-dimensional questionnaire used to measure disability levels across various conditions. Validated in 16 languages across 14 countries, it has adequate internal consistency, construct, and discriminate validity. The tool evaluates a patient’s overall disability under the International Classification of Functioning, Disability and Health (ICF). The WHODAS 2.0 is self-reported and administered to participants aged 18 and above (25).

### Procedure

We included1,986 patients with orthopaedic injuries following road traffic injuries using the Kampala Trauma Score (KTS) classified as mild, moderate and severe to assess injury severity (26, 27). Participants were invited to the hospital to assess their status 2 years post-RTIs. The EQ-5D-5L/VAS questionnaire was used to measure patients’ quality of life. Socioeconomic status was categorized according to the Rwandan government, with categories including impoverished, vulnerable, gainfully employed, employers, and proprietors (28). The primary outcome was quality of life, evaluated using the EQ-5D-5L/VAS scores. Secondary outcomes included demographic data, the KTS score, hospital stay, rehabilitation, return to work, and level of disability, all evaluated through a predesigned questionnaire. Data were collected from patients’ files and assessed during the study.

### Data management, statistical analysis, and ethical consideration

The data were gathered through questionnaires, inputted into a computer using Google Form’s data entry feature, and analysed using R Software. We conducted a descriptive analysis of the patient-reported outcome measure scale EQ-5D-5L and VAS. Categorical variables were summarized using frequencies and percentages, continuous variables with means and standard deviations (SD). We utilized a student’s t-test for comparing continuous variables and the Chi-Square test for nominal (categorical) variables. A multivariate logistic regression was used to examine the relationships between risk factors and EQ-5D-5L score categories. We computed Shannon’s indices to assess the diversity within our population and a *p*-value less than 0.05 was deemed statistically significant.

Our study obtained ethical clearance from the University of Rwanda College of Medicine and Health Sciences Institutional Review Board (18/CMHS IRB/2022). It was authorized by the Rwanda National Research Committee under the Ministry of Health (NHRC/2022/PROT/014). Reference 5535/RBC/2022 created collaboration with the Rwanda Biomedical Centre injury department. Five hospital ethics committees approved our study: CHUK (EC/CHUK/051/2022), CHUB (REC/UTHB/089/2022), RH (313/RRH/DG/2022), KFH (EC/KFH/015/2022), and RMH. All research participants gave written agreement after being told the purpose.

## Results

### Demographic characteristics of the participants vs. EQ-5D-5L VAS score

369 RTOI victims were included (Table 1). 64.5% (238) were CHUK recruits. The participants’ average age was 37.5 ± 11.26 years, with the majority aged 31–50. Young adults (18–30) had the best quality of life (47.6%), while those over 45 the worst (42.6%). Men dominated (74.25%). Both men and women with injuries have similar quality of life, suggesting no gender difference. About 41.73% (154), were in business, while 29% (107) were unemployed. Also, motorcycle accidents caused 61.52% of injuries. Primary school was attended by 41.73% (172). The highest quality of life was reported by university graduates (66.10%). 46.34% (171) lived in Kigali (Table 2). Most participants (61.52%, 227) were from socioeconomic class III (Ubudehe).

**Table 1:** Demographic characteristics of the participants (*n* = 369).

Factors			EQ-5D-5L VAS score							
	Poor (0–40%) *N* = 129		Fair (41–60%) *N* = 109		Good (61–100%) *N* = 131		Test statistics		Total (Factor)	
	*N*	%	*N*	%	*N*		X^2^	*p*	*N*	
Age Mean = 37.57(±11.26)							9.653	0.047		
18–30	30	29.41	24	23.53	48	47.6			102	27.71
31–45	70	35.18	64	32.16	65	32.66			199	53.92
>45	29	42.65	21	30.88	18	26.47			68	18.42
Sex							1.87	0.391		
Male	92	33.58	86	31.39	96	35.04			274	74.25
Female	37	39.96	23	24.21	35	36.84			95	25.75
Hospital of treatment							17.22	0.028		
CHUK	78	32.77	72	30.25	88	36.97			238	64.49
CHUB	14	43.75	8	25.00	10	31.25			32	8.67
RH	10	34.48	13	44.83	6	20.69			29	7.85
RMH	6	20.00	7	23.33	17	56.67			30	8.13
KFH	21	56.67	9	22.50	10	25.00			40	10.84
Level of education							35.318	<0.01		
None	12	42.86	10	35.71	6	21.43			28	7,58
Primary	73	42.44	55	31.98	44	25.58			172	41.73
Secondary	35	31.82	33	30.00	42	38.18			110	29.81
University	9	15.25	11	18.64	39	66.10			59	15.99
Residence							6.36	0.041		
Kigali City	55	32.16	61	35.67	36	21.05			46.5	
Secondary cities	22	23.40	37	39.36	17	18.09			25.5	
Other Districts	18	17.48	43	41.75	28	27.18			28	
Occupation							27.10	0.003		
Farmer	9	29.03	15	48.39	7	22.58			31	8.40
Business	44	28.57	46	29.87	64	41.56			154	41.73
Students	2	40.00	2	20.00	2	40.00			5	1.36
Public service	18	31.03	10	17.24	30	51.72			58	15.72
Informal sector	51	47.66	32	29.91	24	22.43			107	29.00
Retired	5	35.71	5	35.71	4	28.57			14	3.79
Socio-economic status (Ubudehe)							2.53	0.638		
I	10	50.00	5	25.00	5	25.00			20	5.42
II	44	36.07	36	29.51	42	34.43			122	33.06
III	75	33.04	68	29.96	84	37.00			227	61.52
Cause of the injury							10.42	0.034		
Motorcycles	70	29.29	77	32.22	92	38.49			239	64.76
Cars	39	43.82	21	23.60	10	32.58			89	24.11
Others	20	48.78	11	26.83	10	24.39			41	11.11

**Table 2:** Clinical factors vs. EQ-5D-5L VAS score.

Factors	EQ-5D-5L VAS score									
	Poor (0–40%) (*N* = 129)		Fair (41–60%) (*N* = 109)		Good (61–100%) (*N* = 131)		Test statistics		Total (Factors)	
	*N*	%	*N*	%	*N*	%	X^2^	*p*	*N*	%
Kampala Trauma Score (KTS)							24.61	<0.01		
Mild	1	4.55	3	13.64	18	81.82			22	5.96
Moderate	86	34.82	75	30.36	86	34.82			247	66.93
Severe	42	42.00	31	31.00	27	27.00			100	27.10
Diagnosis							30.23	<0.01		
Upper extremity injuries	11	22.92	13	27.08	24	50.00			48	13.01
Lower extremity injuries	74	37.95	61	31.28	24	50.00			195	52.85
Upper and lower extremity injuries	5	25.00	10	50.00	5	25.00			20	5.42
Polytrauma	35	44.87	21	26.92	22	28.21			78	21.14
Soft tissues injuries	4	14.29	4	14.29	20	71.43			28	7.59
Time before management							13.74	0.008		
≤1 day	62	34.25	64	35.36	36	19.89			182	49.32
2–7 days	20	17.24	45	38.79	28	24.14			116	31.44
8–14 days	3	13.04	11	47.83	6	26.09			23	6.23
15–30 days	7	23.33	11	36.67	7	23.33			30	8.13
>30 days	3	16.67	10	55.56	4	22.22			18	4.88
Intervention							47.27	<0.01		
Closed reduction+POP	7	17.50	16	40.00	17	42.50			40	10.84
ORIF	59	38.06	52	33.55	44	28.39			155	42.01
OREF	33	57.89	13	22.81	11	19.30			57	15.45
Amputation	8	66.67	3	25.00	1	8.33			12	3.25
Other	22	20.95	25	23.81	58	55.24			105	28.46
Length of Hospital Stay							45.92	<0.01		
0–7 days	31	20.81	38	25.50	80	53.69			149	40.38
8–14 days	20	36.36	17	30.91	18	32.73			55	14.91
15–30 days	28	39.44	27	38.03	16	22.54			71	19.24
>30 days	50	53.19	27	28.72	17	18.09			94	25.47
Rehabilitation							42.74	<0.01		
Yes	67	28.88	65	28.02	100	43.10			232	62.87
No	62	45.26	44	32.12	31	22.63			137	37.13
Return to work							45.74	<0.01		
Yes	54	22.98	74	31.49	107	45.53			235	63.68
No	75	55.97	35	26.12	24	17.91			134	36.31
Disability compensation							1.99	0.369		
Yes	53	33.97	52	33.33	51	32.69			156	42.27
No	76	35.96	109	29.54	131	35.50			213	57.72

KTS, Kampala Trauma Score; ORIF, Open Reduction Internal Fixation; OREF, Open Reduction External Fixation; POP, Plaster of Paris; LEFS, Lower Extremity Functional Score.

### Clinical factors

52.85% of participants had isolated lower limb injuries and 21.14% polytrauma. The majority had moderate Kampala Trauma Score (KTS) at 66.84%, with mild KTS having a higher success rate. Half of the participants were managed within 1 day, with a mean treatment duration of 30 days. Open Reduction Internal Fixation (ORIF) and Open Reduction External Fixation (OREF) treatments had better outcomes compared to closed reduction +Plaster of Paris (POP). 55.29% were discharged within 14 days, with a mean hospital stay of 30 days. After injury treatment, 37.13% could not undergo rehabilitation, and 36.3% had still not returned to work 2 years after the injury.

### EQ-5D-5L frequencies and proportions reported by dimension and level

**Figure 1:**
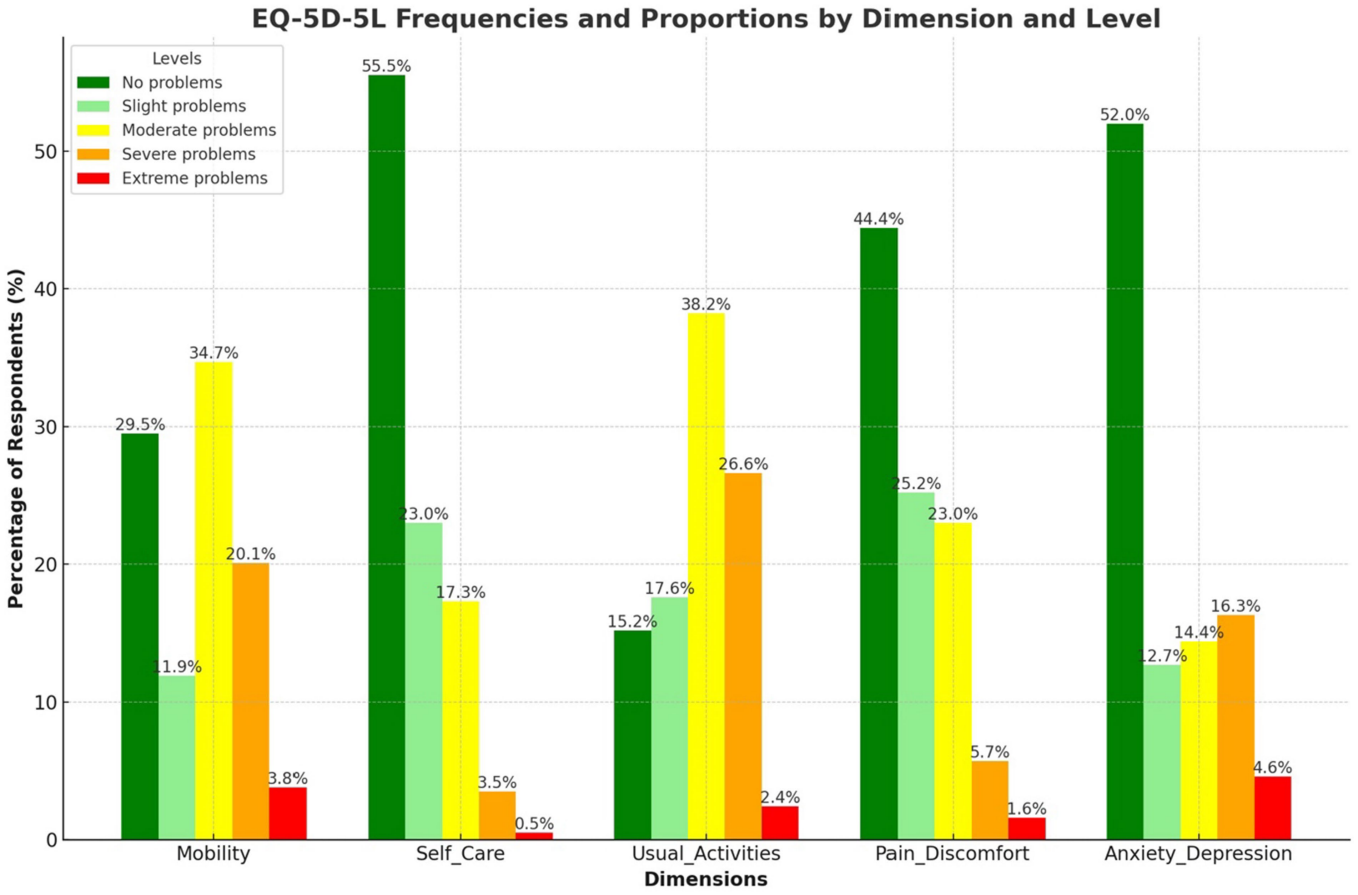
EQ-5D-5L frequencies and proportions reported by dimension and level.

Figure 1 displays 369 individuals’ EQ-5D-5L health scores in mobility, self-care, regular activities, pain/discomfort, and anxiety/depression. Mobility was the most common category with 34.7% having moderate concerns. However, 29.5% reported no mobility concerns. In Self-Care, 55.56% of participants reported no problems, while 23.04% had minor issues. 38.2% had moderate difficulties with daily duties. 44.4% reported no pain or discomfort, whereas 25.2% experienced mild pain. Most individuals (52.0%) did not express anxiety or depression, although 16.3% had severe symptoms.

### Univariate analysis of the predictors and the dimensions of the EQ-5D-5L

**Figure 2:**
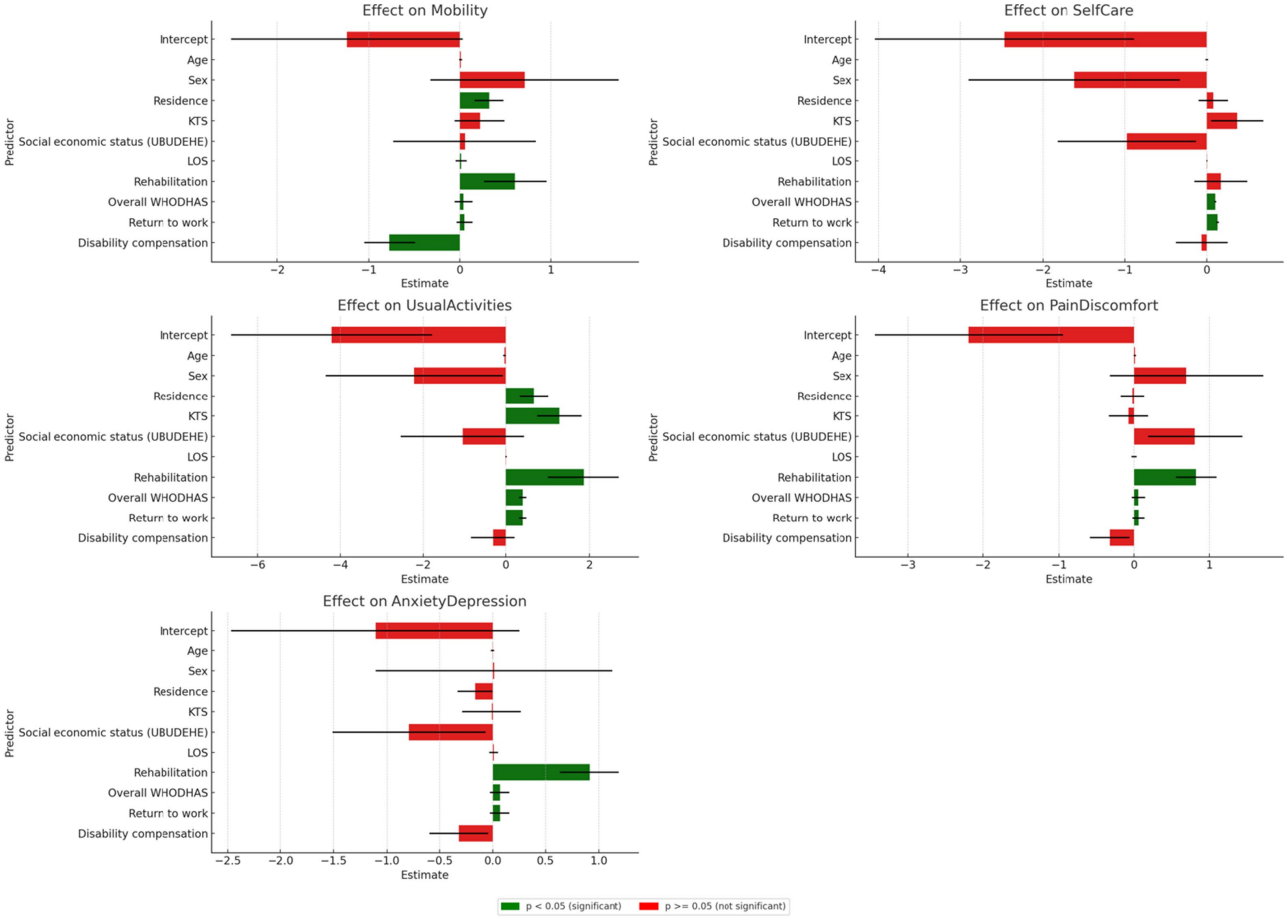
Univariate analysis of the predictors and the dimensions of the EQ-5D-5L.

Figure 2 shows significant correlations between residence, length of stay, rehabilitation, and return to work with mobility, usual activities, pain/discomfort, and anxiety/depression (*p* < 0.005). The WHODHAS scores are highly significant across all dimensions (*p* = 0.000). Return to work is significantly correlated with mobility, self-care, usual activities, pain/discomfort, and anxiety/depression (*p* < 0.005). Additionally, disability compensation is notably correlated with mobility, which in turn is associated with a favourable quality of life. Predictors like rehabilitation and return to work generally show positive associations with good QoL, suggesting potential improvements or lesser negative impacts on the EQ-5D-5L dimensions (*p* < 0.005). While these correlations suggest possible causal relationships, it is important to note that causality could operate in either direction.

### Heatmap of individual profiles of the EQ-5D-5L

**Figure 3:**
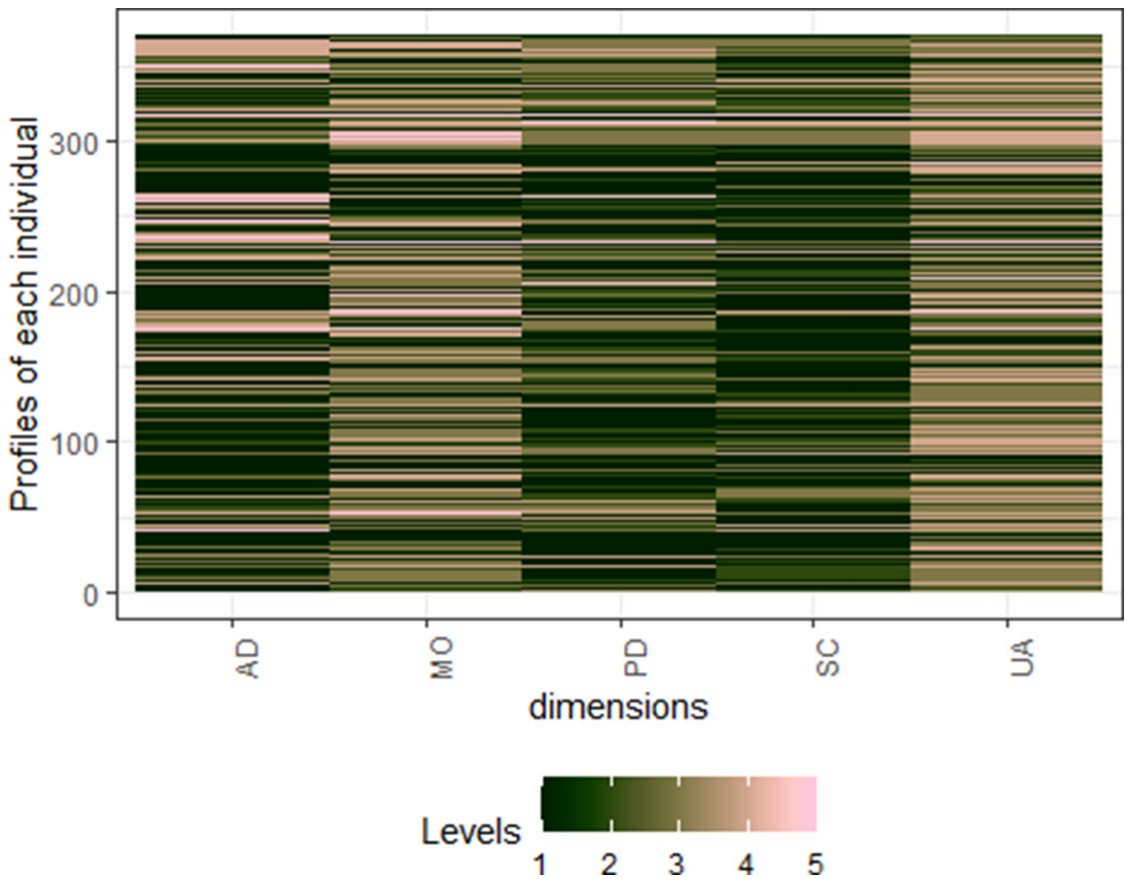
Heatmap of individual profiles of the EQ-5D-5L. AD, Anxiety and Depression; MO, Mobility; PD, Pain and discomfort; SC, Self-care; UA, Usual activities. Level 1: No problem, Level 2: Slight problem, Level 3: Moderate problems, Level 4: Severe problems, and Level 5: Extreme problems.

The heatmap shows a diverse quality of life among Rwandan road traffic orthopaedic injury survivors. The most common health state is ‘11111’, indicating no issues, but moderate to extreme difficulties are present, especially in mobility and pain/discomfort dimensions. This reflects a range of individual health outcomes across five health dimensions: Mobility, Self-Care, Usual Activities, Pain/Discomfort, and Anxiety/Depression. This highlights the need for tailored healthcare and rehabilitation services (Figure 3).

### Multinomial logistics regression EQ-5D-5L/VAS and predictors

The multinomial logistic regression analysis reveals that various factors, including education, cause and type of injury, rehabilitation, physical function, and level of disability, significantly influence the quality of life of survivors of road traffic orthopaedic injuries in Rwanda (Table 3). Higher levels of education are significantly associated with better quality of life outcomes, with each additional unit increasing the odds by 66%. The specific cause of injury has a slight but statistically significant association with quality of life, with a 10% decrease in the odds of better outcomes for certain causes compared to others. The type of surgical or medical intervention received in the operating theatre is also significantly associated with quality-of-life outcomes. Higher levels of disability are strongly associated with poorer quality of life outcomes, almost doubling the odds of worse quality of life.

**Table 3:** Multinomial logistics regression EQ-5D-5L/VAS and predictors.

EQ-5-5D-5L/VAS	Predictors	OR	CI	*p*-value
1	Level of Education	1.66	1.32–2.10	<0.01
2	Cause of injury	0.90	0.83–0.98	0.048
3	Type of intervention in theatre	1.22	1.07–1.40	0.003
3	Rehabilitation	2.41	1.28–1.60	<0.01
4	Return to work	0.235	0.30–0.71	0.001
5	LEFS	1.05	1.00–1.03	0.001
6	Level of Disability (WHODAS2.0 score)	1.96	1.95–1.96	<0.01

### Health state density curve and health state density index

**Figure 4:**
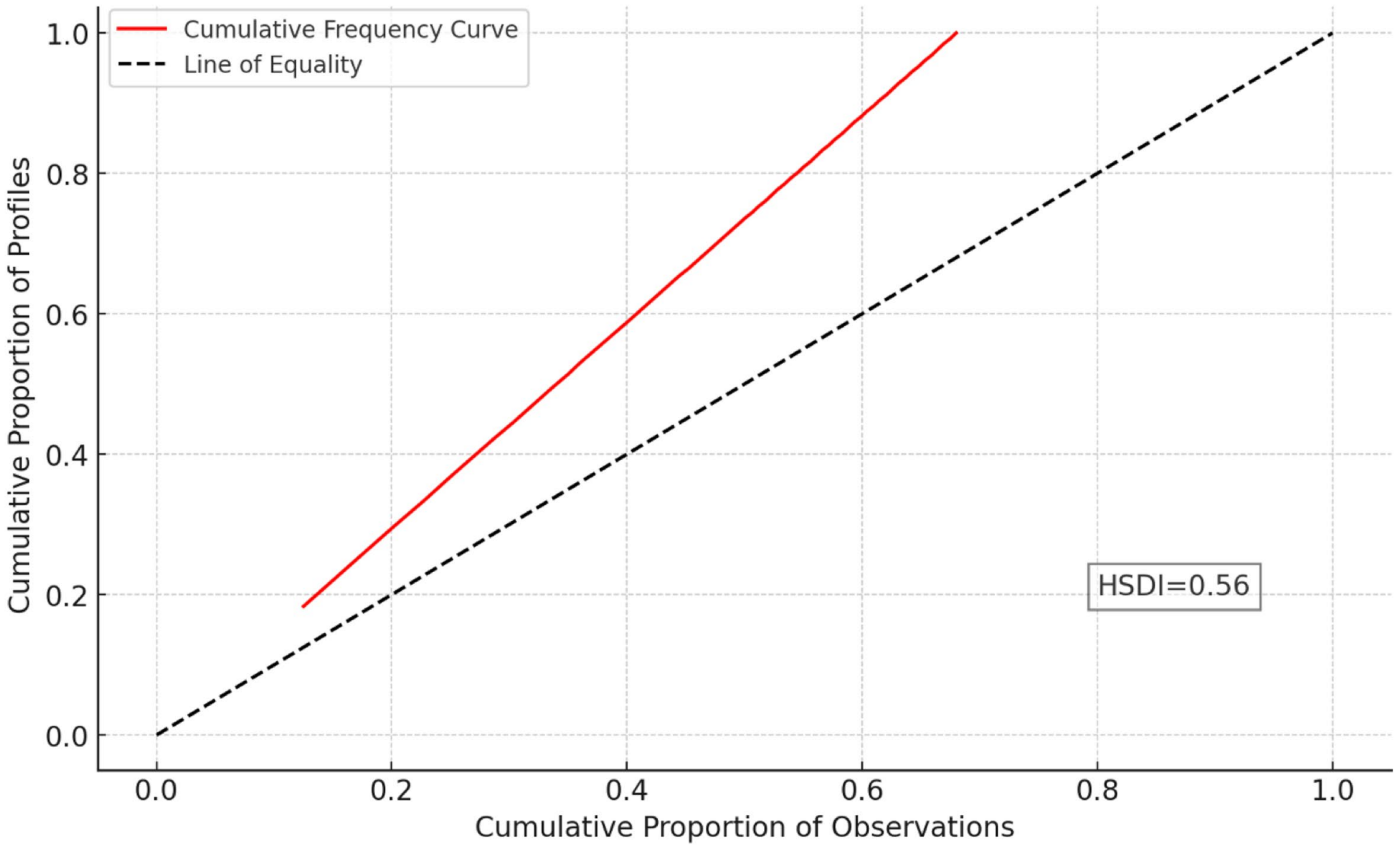
Health State Density Curve (HSDC) and Health State Density Index (HSDI).

Figure 4 represent the cumulative frequency curve which is red, and the dashed black line is the line of equality, indicating a perfect health state distribution. This image’s HSDC has a similar curve, with the Health State Density Index (HSDI) between the curve and the line of equality. The displayed curve depicts the vast variety of health statuses with variable frequencies in the text data. The plot’s HSDI value (0.56) suggests modest health state inequality among Rwandan road traffic orthopaedic injury survivors, with a concentration of persons in a few health states.

### Shannon’s indices of road traffic orthopaedic injury survivors

**Figure 5:**
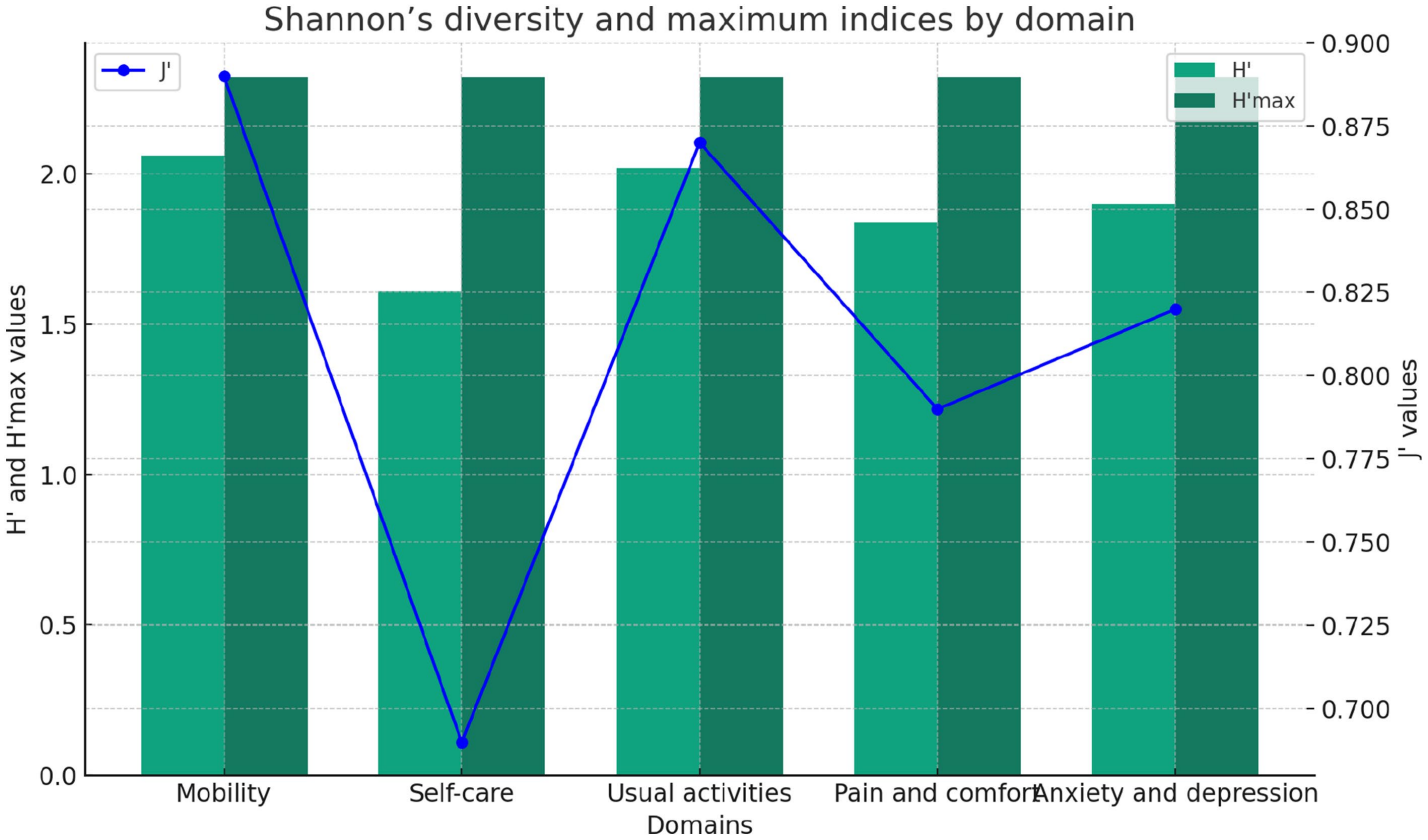
Shannon’s indices of road traffic orthopaedic injury survivors.

Figure 5 shows Shannon’s indices for Rwandan road traffic orthopaedic injury survivors’ quality of life. Diversity—the distribution of responses across levels or categories—is measured by these indices. The observed diversity (H´) is moderate, with higher values indicating greater diversity. The observed diversity is 6.68, while the maximum possible diversity (H’max) is 11.61. The J´ (0.58) measure of response distribution equitability ranges from 0.69 to 0.87, indicating moderate evenness. Mobility’s moderate diversity is 2.06, maximum potential diversity is 2.32, and evenness is 0.89. Low diversity of 1.61, maximum potential diversity of 2.32, and evenness of 0.69 characterize self-care. Moderate diversity is 2.02, maximum potential diversity is 2.32, and evenness is 0.87 for usual activities. Pain and comfort have 1.84 moderate diversity, 2.32 maximum potential diversity, and 0.79 evenness. Anxiety and depression have 1.9 moderate diversity, 2.32 maximum diversity, and 0.82 evenness.

Shannon’s indices show moderate diversity in experiences for Rwandan road traffic orthopaedic injury survivors, with mobility showing high diversity and evenness. Self-care has lower diversity but reasonable evenness. Usual activities, pain, comfort, anxiety, and depression have moderate diversity and high evenness, reflecting a nuanced picture of post-injury quality of life.

## Discussion

We evaluated the quality of life of survivors following road traffic orthopaedic injuries (upper limbs and lower limbs injuries) in Rwanda. Factors such as age, gender, level of education, hospital of treatment, residence, occupation, socio-economic status, and cause of the injury were considered.

Our results show that most survivors were male, with a mean age of 37.57 years which is like other studies on road traffic injuries in LMICs (29, 30). The quality-of-life post RTOI in Rwanda is significantly influenced by various demographic factors, including young age (3, 10, 18–42), education level from secondary school to university, urban residence location of the patients, victim’s occupation (business), male sex, and socio-economic status class III. Enhanced resilience is fostered by younger people, persons with a university education, and job cultures that provide support. Health-related quality of life, or HRQOL, is a problem not just in Rwanda; studies have shown that clinical aspects during hospitalization and after release are critical components of overall HRQoL (3, 31).

Half of the injured participants received medical treatment within a day. Preventing complications and improving patient outcomes requires prompt intervention. Their average treatment duration was 30 days, indicating prolonged medical care. Due to the complexity and severity of road traffic orthopaedic injuries, extensive treatment and rehabilitation are needed to maximize recovery and minimize long-term disabilities (15, 32, 33). Femur shaft, distal femur, tibia plateau, ankle, Pilon, and bimalleolar fractures were the main lower limb injuries. According to the literature, knee and ankle fractures always result in long hospital stays and permanent disabilities (34, 35). Such findings emphasize the importance of timely and adequate medical intervention in improving road traffic orthopaedic injury survivors’ quality of life (36, 37).

Although there are alternative methods available, the EQ-5D-5L tool has gained favor in measuring HRQoL, particularly in trauma patients (10). We have found that the EQ-5D-5L health scores reveal that mobility is the most common concern among individuals recovering from road traffic orthopaedic injuries. Mobility issues are often prominent, impacting daily activities and overall well-being. Self-care difficulties are less prevalent, with over half of participants reporting no problems with self-care. Regular activities are also affected, with more than a third experiencing moderate difficulties. Pain and discomfort are prevalent, but not all participants reported issues.

The psychological impact of orthopaedic injuries is significant, with a significant proportion experiencing severe anxiety or depression. Zahra Emrani et al. (22) in Iran have found that mobility, self-care, and usual activity dimensions were conserved, and many patients had psychological problems (22). Our study emphasizes the importance of addressing both physical and psychological well-being to optimize long-term health outcomes following road traffic orthopaedic injuries.

The findings of our study demonstrate that patient outcomes are influenced by a variety of variables, including mobility, regular activities, pain/discomfort, and anxiety/depression. The factors that have a substantial influence on the EQ-5D-5L dimensions of our participants include residency, duration of hospital stay, rehabilitation, return to work, and disability compensation. Rehabilitation improves mobility, self-care, and overall quality of life. Return to work is crucial for health and well-being, and disability compensation can enhance physical functioning. Several studies assessing the health-Related Quality of Life (HRQoL) have consistently revealed similar characteristics that influence several aspects. However, these studies used various methods to evaluate the Quality of Life (QoL) in Road Traffic Injuries (RTI) (38–40).

In the context of Rwanda, our research on RTOI revealed a heterogeneous distribution of self-perceived health-related quality of life among individuals who had experienced the injury 2 years before. The majority rated their quality of life within the 41–60 range, indicating moderate satisfaction and well-being. However, a smaller percentage reported extreme scores, indicating polarization in their perceived quality of life. These findings highlight the diverse experiences and challenges faced by survivors of RTOI in Rwanda affecting their mental health. In their review, Mahla Babaie et al. (2023) discovered that even in high-income nations with a high prevalence of mental illness, the quality-of-life following RTI results in lifelong handicap (41).

We found a strong correlation between clinical, socioeconomic, and demographic characteristics and patients’ self-rated health after road traffic injuries. Young age, marital status, urban residence, hospital stay duration below 2 weeks, return to work status post-therapy, and low disability level were significant predictors of QoL. Rissanen et al. (2020) identified age and gender as the primary determinants of HRQoL in their study, while also highlighting the significance of clinical and socio-economic variables (42). Our study emphasizes the importance of considering a range of variables in assessing and addressing patients’ well-being, emphasizing the need for a comprehensive approach to patient assessment and treatment.

Our findings show that high level of education, theatre intervention (ORIF and OREF), LEFS scores, injury aetiology, rehabilitation, and disability levels had a favourable influence on health outcomes in patients recovering from orthopaedic injuries (upper limbs and lower limbs injuries) in Rwanda, as seen in previous studies (3, 19, 22, 43, 44). The findings underscore the significance of specific treatments that target education, rehabilitation, and functional status to enhance health outcomes in RTOI victims.

Our data, analysed using Shannon’s indices, indicate modest variance in HRQoL responses among RTOI victims. There is moderate variability in mobility, normal activities, pain, comfort, anxiety, and sadness, whereas self-care shows reduced diversity. The results emphasize the need of considering different aspects of well-being in rehabilitation and support programs. The research conducted by Nena Kruithof et al. (2020) yielded similar results, examining the impact of trauma on health status and psychological outcomes in the Netherlands region.

Our findings revealed a higher prevalence of anxiety and depression compared to other domains (45). Additional study might aid in creating specific interventions to enhance HRQoL outcomes in this group. Using the Health State Density Curve (HSDC) and Health State Density Index (HSDI), we found a modest health state inequality among Rwandan road traffic orthopaedic injury survivors, with a concentration in a few health states. Dipnall et al. (2021) in their study among children and adult have found almost the same where some predictors of health dominant more than others (46).

Our study has some limitations. The cross-section design may introduce recall bias, and self-reported measures may not fully capture the complexity of individuals’ experiences. The focus on survivors seeking medical care may overlook those who did not and those who succumbed to their injuries, leading to selection bias. We may not have accounted for all relevant variables influencing quality of life outcomes. The generalizability of the findings may be limited to similar settings with similar healthcare systems and injury profiles. Addressing these limitations is important for advancing understanding and informing targeted interventions. Our study has produced new insights highlighting the necessity for comprehensive interventions. It emphasizes the significance of education and rehabilitation, as well as utilizing Shannon’s indices analysis for customized interventions. Although limited, the study provides valuable insights into the challenges faced by survivors.

Based on the study findings, we recommend that policymakers should improve rehabilitation services by modernizing facilities, providing specialized training, and integrating them into primary healthcare. Mental health care should be integrated into routine treatment through screening, training in first aid, and accessible support services. Social and economic reintegration programs should include vocational training, job placement, social support networks, and financial assistance. Rwandan psychological support should involve community-based initiatives, training in first aid, and peer counselling. Culturally sensitive interventions and educational campaigns are crucial for promoting positive attitudes towards rehabilitation and mental health care.

## Conclusion

Our study on the quality of life of survivors following Road Traffic Orthopaedic Injuries in Rwanda highlights the importance of addressing both physical and psychological well-being. Factors such as age, education level, residence, occupation, socio-economic status, rehabilitation, level of disability and return to work significantly influenced the quality-of-life post-RTOI. Regaining function was the most common concern among survivors, emphasizing the need for comprehensive interventions targeting education, rehabilitation, and functional status. Shannon’s indices showed modest variance in health-related quality of life responses among survivors, emphasizing the need for a holistic approach to patient assessment and treatment.

## Data availability statement

The datasets presented in this study can be found in online repositories. The names of the repository/repositories and accession number(s) can be found in the article/supplementary material.

## Ethics statement

The studies involving humans were approved by University of Rwanda College of Medicine and Health Sciences Institutional Review Board (18/CMHS IRB/2022). Our research was authorized by the Rwanda National Research Committee under the Ministry of Health (NHRC/2022/PROT/014). Reference 5,535/RBC/2022 created a collaboration with the Rwanda Biomedical Center injury department. Five hospital ethics committees approved our study: CHUK (EC/CHUK/051/2022), CHUB (REC/UTHB/089/2022), RH (313/RRH/DG/2022), KFH (EC/KFH/015/2022), and RMH. All research participants gave written agreement after being told the purpose. The studies were conducted in accordance with the local legislation and institutional requirements. The participants provided their written informed consent to participate in this study.

## Author contributions

JA: Conceptualization, Data curation, Formal Analysis, Funding acquisition, Investigation, Methodology, Project administration, Resources, Software, Supervision, Validation, Visualization, Writing – original draft, Writing – review & editing. DT: Conceptualization, Data curation, Formal Analysis, Funding acquisition, Methodology, Project administration, Resources, Supervision, Validation, Visualization, Writing – original draft, Writing – review & editing. JS: Conceptualization, Data curation, Formal Analysis, Funding acquisition, Investigation, Methodology, Project administration, Resources, Software, Supervision, Validation, Visualization, Writing – original draft, Writing – review & editing. GU: Conceptualization, Data curation, Formal Analysis, Funding acquisition, Investigation, Methodology, Project administration, Resources, Software, Supervision, Validation, Visualization, Writing – original draft, Writing – review & editing. GB: Conceptualization, Data curation, Formal Analysis, Funding acquisition, Investigation, Methodology, Project administration, Resources, Software, Supervision, Validation, Visualization, Writing – original draft, Writing – review & editing. SP: Conceptualization, Data curation, Formal Analysis, Funding acquisition, Investigation, Methodology, Project administration, Resources, Software, Supervision, Validation, Visualization, Writing – original draft, Writing – review & editing. AS: Conceptualization, Data curation, Formal Analysis, Funding acquisition, Investigation, Methodology, Project administration, Resources, Software, Supervision, Validation, Visualization, Writing – original draft, Writing – review & editing.

## References

[1] Organização Mundial da Saúde. Global status Report on road. France: World Health Organization (2018). 20 p.

[2] Kovačević J Miškulin M Matić Ličanin M Barać J Biuk D Palenkić H. Quality of life in road traffic accident survivors. Zdr Varst. (2020) 59:202–10. doi: 10.2478/sjph-2020-0026, PMID: 33133276 PMC7583431

[3] Vu HM Dang AK Tran TT Vu GT Truong NT Nguyen CT. Health-related quality of life profiles among patients with different road traffic injuries in an urban setting of Vietnam. Int J Environ Res Public Health. (2019) 16:1462. doi: 10.3390/ijerph16081462, PMID: 31022979 PMC6517995

[4] Rissanen R Berg HY Hasselberg M. Quality of life following road traffic injury: a systematic literature review. Accid Anal Prev. (2017) 108:308–20. doi: 10.1016/j.aap.2017.09.013, PMID: 28942041

[5] Haghighi MRR Sayari M Ghahramani S Lankarani KB. Social, economic, and legislative factors and global road traffic fatalities. BMC Public Health. (2020) 20:1413. doi: 10.1186/s12889-020-09491-x, PMID: 32943034 PMC7646406

[6] Neagu OA. Insights on the estimate of costs in road traffic accidents. Rev Med Chir Soc Med Nat. (2020) 121:624–30.

[7] Üzümcüoǧlu Y Özkan T Lajunen T Morandi A Orsi C Papadakaki M. Life quality and rehabilitation after a road traffic crash: a literature review. Transp Res Part F Traffic Psychol Behav. (2016) 40:1–13. doi: 10.1016/j.trf.2016.02.002

[8] Faux SG Kohler F Mozer R Klein LA Courtenay S D’Amours SK. The ROARI project - road accident acute rehabilitation initiative: a randomised clinical trial of two targeted early interventions for road-related trauma. Clin Rehabil. (2015) 29:639–52. doi: 10.1177/0269215514552083, PMID: 25413170

[9] Monárrez-Espino J Laflamme L Berg HY. Measuring and assessing risk of quality of life loss following a road traffic injury: a proposed methodology for use of a composite score. Accid Anal Prev. (2018) 115:151–9. doi: 10.1016/j.aap.2018.02.009, PMID: 29573601

[10] Sakran JV Ezzeddine H Schwab CW Bonne S Brasel KJ Burd RS. Proceedings from the consensus conference on trauma patient-reported outcome measures. J Am Coll Surg. (2020) 230:819–35. doi: 10.1016/j.jamcollsurg.2020.01.032, PMID: 32201197

[11] Marquez P. (2013). The challenge of non-communicable diseases and road traffic injuries. World Bank Report.

[12] Alemany R Ayuso M Guillén M. Impact of road traffic injuries on disability rates and long-term care costs in Spain. Accid Anal Prev. (2013) 60:95–102. doi: 10.1016/j.aap.2013.08.016, PMID: 24036315

[13] Ingabire A Petroze RT Calland F Okiria JC Byiringiro JC. Profile and economic impact of motorcycle injuries treated at a university referral hospital in Kigali, Rwanda. Med J. (2015) 72:5–11.

[14] Allen Ingabire J Stewart A Sagahutu JB Urimubenshi G Bucyibaruta G Pilusa S. Prevalence and levels of disability post road traffic orthopaedic injuries in Rwanda. Afr J Disabil. (2024) 13:13. doi: 10.4102/ajod.v13i0.1251 PMC10844983 38322752

[15] Allen Ingabire J Stewart A Uwakunda C Mugisha D Sagahutu JB Urimubenshi G. Factors affecting social integration after road traffic orthopaedic injuries in Rwanda. Front Rehabil Sci. (2024) 4:1287980. doi: 10.3389/fresc.2023.1287980 38293289 PMC10825670

[16] Rosenberg GM Stave C Spain DA Weiser TG. Patient-reported outcomes in trauma: a scoping study of published research. Trauma Surg Acute Care Open. (2018) 3:1–6. doi: 10.1136/tsaco-2018-000202 PMC6135428 30234168

[17] De AB FD Cividini FR. Challenges faced by nursing in the emergency room for the care of trauma victims: an integrative literature review. Res Soc Dev. (2023) 12:e8512541504. doi: 10.33448/rsd-v12i5.41504

[18] Biz C Buffon L Marin R Petrova N. Orthopaedic nursing challenges in poly-traumatised patient management: a critical analysis of an Orthopaedic and trauma unit. Int J Orthop Trauma Nurs. (2016) 23:60–71. doi: 10.1016/j.ijotn.2016.04.003, PMID: 27561247

[19] Gane EM Plinsinga ML Brakenridge CL Smits EJ Aplin T Johnston V. The impact of musculoskeletal injuries sustained in road traffic crashes on work-related outcomes: A systematic review. Int J Environ Res Public Health. (2021) 18. doi: 10.3390/ijerph182111504, PMID: 34770019 PMC8582890

[20] Fincham JE Draugalis JR. The importance of survey research standards. Am J Pharm Educ. (2013) 77:7–10. doi: 10.5688/ajpe7714 23460755 PMC3578336

[21] Rabin R De Charro F. EQ-5D: A measure of health status from the EuroQol group. Ann Med. (2001) 33:337–43. doi: 10.3109/07853890109002087 11491192

[22] Emrani Z Akbari Sari A Zeraati H Olyaeemanesh A Daroudi R. Health-related quality of life measured using the EQ-5D-5 L: population norms for the capital of Iran. Health Qual Life Outcomes. (2020) 18. doi: 10.1186/s12955-020-01365-5 PMC7183694 32334604

[23] Payakachat Ali Tilford (2015) Can the EQ-5D detect meaningful change a systematic review. 10.1007/s40273-015-0295-6 PMC4609224 26040242

[24] Foundation ER (2020). User Guide User Guide. EuroQol Research Foundation 2021. 169–232 p.

[25] Svanborg C Amer A Nordenskjöld A Ramklint M Söderberg P Tungström S. Evidence for validity of the Swedish self-rated 36-item version of the World Health Organization disability assessment schedule 2.0 (WHODAS 2.0) in patients with mental disorders: a multi-Centre cross-sectional study using Rasch analysis. J Patient Rep Outcomes. (2022) 6:45. doi: 10.1186/s41687-022-00449-8, PMID: 35526195 PMC9081069

[26] Zziwa S Babikako H Kwesiga D Kobusingye O Bentley JA Oporia F. Prevalence and factors associated with utilization of rehabilitation services among people with physical disabilities in Kampala, Uganda. A descriptive cross sectional study. BMC Public Health. (2019) 19:1–11. doi: 10.1186/s12889-019-8076-3 31881994 PMC6935194

[27] Haac B Varela C Geyer A Cairns B Charles A. The utility of the Kampala trauma score as a triage tool in a sub-Saharan African trauma cohort. World J Surg. (2015) 39:356–62. doi: 10.1007/s00268-014-2830-6 25315093

[28] Sabates-Wheeler R Yates S Wylde E Gatsinzi J. Challenges of measuring graduation in Rwanda. IDS Bull. (2015) 46:103–14. doi: 10.1111/1759-5436.12133

[29] Mousazadeh Y Sadeghi-Bazargani H Janati A Poustchi H Zakeri R Shafiee-Kandjani A. Functional consequences of road traffic injuries: preliminary results from PERSIAN traffic cohort (PTC). Trauma Mon. (2021) 26:294–304. doi: 10.30491/TM.2021.289262.1314

[30] Gheshlaghi LA Shari H. (2020). Quality of life after motorcycle Tra c injuries: A cohort study in northwest of Iran. 1–14.

[31] Afolabi OJ Kolawole TG. Road traffic crashes in Nigeria: causes and consequences. Int J Shipp Transport Logist. (2017) 17:40–9.

[32] Wu J Faux SG Estell J Wilson S Harris I Poulos CJ. Early rehabilitation after hospital admission for road trauma using an in-reach multidisciplinary team: a randomised controlled trial. Clin Rehabil. (2017) 31:1189–200. doi: 10.1177/0269215517694462, PMID: 28786337

[33] D’Alleyrand JCG O’Toole RV. The evolution of damage control orthopedics. Current evidence and practical applications of early appropriate care. Orthop Clin N Am. (2013) 44:499–507. doi: 10.1016/j.ocl.2013.06.004 24095066

[34] Biz C Angelini A Zamperetti M Marzotto F Sperotto SP Carniel D. Medium-long-term radiographic and clinical outcomes after surgical treatment of intra-articular tibial pilon fractures by three different techniques. Biomed Res Int. (2018) 2018:1–12. doi: 10.1155/2018/6054021, PMID: 29687005 PMC5852840

[35] Biz C Maso G Gambato M Belluzzi E Pozzuoli A Favero M. Challenging surgical treatment of displaced articular Tibial plateau fractures: do early knee radiographic features have a predictive value of the mid-term clinical functional outcomes? Orthop Surg. (2019) 11:1149–62. doi: 10.1111/os.12577, PMID: 31755217 PMC6904635

[36] Bach JA Leskovan JJ Scharschmidt T Boulger C Papadimos TJ Russell S. The right team at the right time - multidisciplinary approach to multi-trauma patient with orthopedic injuries. Int J Crit Illn Inj Sci. (2017) 7:32–7. doi: 10.4103/IJCIIS.IJCIIS_5_17, PMID: 28382257 PMC5364767

[37] Guerado E Bertrand ML Cano JR Cerván AM Galán A (2019). View of damage control in orthopedics and traumatology. 10.5312/wjo.v10.i1.1 PMC6354106 30705836

[38] Rezai M. (2018). Work ability and return to work following a traffic injury. ProQuest Dissertations and Theses [Internet]. 261. Available from: http://proquest.umi.com/login/athens?url=https://search.proquest.com/docview/2080287281?accountid=11979%0Ahttps://onesearch.lancaster-university.uk/openurl/44LAN/44LAN_services_page?url_ver=Z39.88-2004&rft_val_fmt=info:ofi/fmt:kev:mtx:dissertation&genre=d (Accessed 29 February 2024).

[39] Gray SE Hassani-Mahmooei B Kendall E Cameron ID Kenardy J Collie A. Factors associated with graduated return to work following injury in a road traffic crash. J Transp Health. (2018) 10:167–77. doi: 10.1016/j.jth.2018.07.006

[40] Pélissier C Fort E Fontana L Charbotel B Hours M. Factors associated with non-return to work in the severely injured victims 3 years after a road accident: a prospective study. Accid Anal Prev. (2017) 106:411–9. doi: 10.1016/j.aap.2017.06.020, PMID: 28728063

[41] Babaie M Joulani M Ranjbar Hameghavandi MH Asgardoon MH Nojomi M O’Reilly GM. Risk of permanent medical impairment after road traffic crashes: a systematic review. Chin J Traumatol. (2023) 26:267–75. doi: 10.1016/j.cjtee.2022.11.002, PMID: 36577609 PMC10533538

[42] Rissanen R Ifver J Hasselberg M Berg HY. Quality of life following road traffic injury: the impact of age and gender. Qual Life Res. (2020) 29:1587–96. doi: 10.1007/s11136-020-02427-3, PMID: 31960212 PMC7253518

[43] Huang S Dipnall JF Gabbe BJ Giummarra MJ. Pain and mental health symptom patterns and treatment trajectories following road trauma: a registry-based cohort study. Disabil Rehabil. (2022) 44:8029–41. doi: 10.1080/09638288.2021.2008526, PMID: 34871122

[44] Gopinath B Jagnoor J Kifley A Dinh M Craig A Cameron ID. Predictors of health-related quality of life after non-catastrophic injury sustained in a road traffic crash. Ann Phys Rehabil Med. (2020) 63:280–7. doi: 10.1016/j.rehab.2019.10.001, PMID: 31689539

[45] Kruithof N Polinder S de Munter L van de Ree CLP Lansink KWW de Jongh MAC. Health status and psychological outcomes after trauma: a prospective multicenter cohort study. PLoS One. (2020) 15:e0231649. doi: 10.1371/journal.pone.0231649, PMID: 32315373 PMC7173764

[46] Dipnall JF Rivara FP Lyons RA Ameratunga S Brussoni M Lecky FE. Health-related quality of life (Hrqol) outcomes following injury in childhood and adolescence using euroqol (eq-5d) responses with pooled longitudinal data. Int J Environ Res Public Health. (2021) 18:10156. doi: 10.3390/ijerph181910156, PMID: 34639458 PMC8507627

